# Nursing Perspectives on the Impacts of COVID-19: Social Media Content Analysis

**DOI:** 10.2196/31358

**Published:** 2021-12-10

**Authors:** Ainat Koren, Mohammad Arif Ul Alam, Sravani Koneru, Alexa DeVito, Lisa Abdallah, Benyuan Liu

**Affiliations:** 1 Solomont School of Nursing University of Massachusetts Lowell Lowell, MA United States; 2 Department of Computer Science University of Massachusetts Lowell Lowell, MA United States

**Keywords:** mental health, information retrieval, coronavirus, COVID-19, nursing, nurses, health care workers, pandemic, impact, social media analytics

## Abstract

**Background:**

Nurses are at the forefront of the COVID-19 pandemic. During the pandemic, nurses have faced an elevated risk of exposure and have experienced the hazards related to a novel virus. While being heralded as lifesaving heroes on the front lines of the pandemic, nurses have experienced more physical, mental, and psychosocial problems as a consequence of the COVID-19 outbreak. Social media discussions by nursing professionals participating in publicly formed Facebook groups constitute a valuable resource that offers longitudinal insights.

**Objective:**

This study aimed to explore how COVID-19 impacted nurses through capturing public sentiments expressed by nurses on a social media discussion platform and how these sentiments changed over time.

**Methods:**

We collected over 110,993 Facebook discussion posts and comments in an open COVID-19 group for nurses from March 2020 until the end of November 2020. Scraping of deidentified offline HTML tags on social media posts and comments was performed. Using subject-matter expert opinions and social media analytics (ie, topic modeling, information retrieval, and sentiment analysis), we performed a human-in-a-loop analysis of nursing professionals’ key perspectives to identify trends of the COVID-19 impact among at-risk nursing communities. We further investigated the key insights of the trends of the nursing professionals’ perspectives by detecting temporal changes of comments related to emotional effects, feelings of frustration, impacts of isolation, shortage of safety equipment, and frequency of safety equipment uses. Anonymous quotes were highlighted to add context to the data.

**Results:**

We determined that COVID-19 impacted nurses’ physical, mental, and psychosocial health as expressed in the form of emotional distress, anger, anxiety, frustration, loneliness, and isolation. Major topics discussed by nurses were related to work during a pandemic, misinformation spread by the media, improper personal protective equipment (PPE), PPE side effects, the effects of testing positive for COVID-19, and lost days of work related to illness.

**Conclusions:**

Public Facebook nursing groups are venues for nurses to express their experiences, opinions, and concerns and can offer researchers an important insight into understanding the COVID-19 impact on health care workers.

## Introduction

### Background

Nursing is an occupation with unique potential for exposure to environmental and occupational hazards in the work setting. Nurses confront potential exposure to infectious diseases, toxic substances, stress, back injuries, and radiation [1]. The COVID-19 epidemic poses a unique, health risk situation that is rapidly evolving [2]. The American Nurses Association Code of Ethics (2015) states that the nursing profession’s nonnegotiable ethical practice standard, according to Provision 2 of the Code, is that “the nurse’s primary commitment is to the patient.” Provision 5 of the Code states that “the nurse owes the same duty to protect themselves” [3]. These two equal obligations can be in conflict during pandemics, when nurses must continually care for critically ill infectious patients under extreme circumstances, including insufficient or inadequate resources and uncontained contagious diseases.

Professional nurses historically bring compassionate competent care to disaster responses but are faced with challenges to provide care when the nature of their work puts them at increased risk. Nurses struggle with feeling physically unsafe in the disaster response situation, such as in times of scarce resources where supplies of personal protective equipment (PPE) may be inadequate [4]. Nurses are concerned about professional, ethical, and legal protection when asked to provide care in such high-risk situations, such as the COVID-19 pandemic. According to DeWolfe, disasters such as the COVID-19 pandemic impact those who experience them psychologically and socially [5]. Whether one considers the COVID-19 pandemic a human-caused or natural disaster, the human effects of living through such an experience are significant, especially when exposure to such a disaster is felt personally. For example, nurses working on the front lines throughout the COVID-19 pandemic have felt a direct effect of this disaster and, therefore, could experience an unusually large number of psychological and social reactions to this experience [5]. DeWolfe explains that high-exposure survivors, such as nurses and other health care workers, could experience a range of effects, such as anxiety, depression, sadness, posttrauma symptoms, somatic symptoms, and substance abuse [5].

Researchers around the world who have been examining the psychological impact on nurses and other health care workers as a result of the COVID-19 pandemic have shown that nurses and other health care workers are experiencing high anxiety and fear, especially as these relate to concerns of infecting family members, being unable to socialize, and transmission of COVID-19 in their work settings [6]. A cross-sectional descriptive analysis of 204 COVID-19–infected health care workers showed that not only does the lack of PPE put nurses at risk of contracting COVID-19 from patients, but the lack of compliance from fellow employees to wear masks and practice social distancing, especially during breaks, puts nurses at risk [6]. An et al found that depressive symptoms among emergency department nurses in China were common, and those reporting higher depressive symptoms also reported lower quality of life [7].

Hu et al examined frontline nurses in Wuhan, China; their findings demonstrated that nurses experienced moderate to high levels of anxiety, depression, burnout, and fear, along with reporting having one or more skin lesions [8,9]. Nurses are also facing ethical dilemmas, such as which patients to prioritize and who should receive ventilation because of a lack of a sufficient number of ventilators [10] as well as moral distress related to uncertainty about their skills to tackle the virus [10]. Qualitative studies have demonstrated that nurses in China who were providing direct care to patients with COVID-19 experienced a range of both positive and negative emotions [11]. Liu et al identified key themes that stress the emotional toll being experienced by nurses, specifically related to feelings of facing challenges and danger, fear of being infected, exhaustion, and stress [12]. Along with these feelings, nurses also expressed their strong sense of duty and responsibility for being a health care provider during this pandemic, along with the hope that the epidemic would be overcome [12].

Sun et al found that in the early weeks of dealing with the pandemic, nurses primarily experienced negative emotions, such as fatigue, discomfort, helplessness, fear, and anxiety [11]; however, with time working in the setting and with knowledge growth of the care they provided, nurses expressed many positive emotions, such as those focusing on coping and self-care, confidence in their self-prevention of contracting COVID-19, and happiness gained from their patients’ respect and from their family and team support. The stress of working with patients with COVID-19 carries over into the daily life of nurses, as they feel isolated from family and friends as well as having their children’s caregivers quit because of fear of infection and being unable to attend funerals of loved ones [6]. Some nurses became frustrated as they found themselves out of work for the first time and wished they could do more to help [10].

### Gap in Knowledge

Various forms of media have played a major role in the COVID-19 pandemic, being major sources of information for the public. However, these media sources have presented contradictory opinions and viewpoints about the virus, causing some to take the virus less seriously than others, leading to more distress for nurses [10]. The science of understanding health-related information that is distributed via social media to inform public health and public policy, known as infodemiology, has been particularly useful for identifying disease outbreak patterns and studying public perceptions of various diseases. Analysis of health event data posted on social media platforms not only provides firsthand evidence of health event occurrences but also enables faster access to real-time information that can help health professionals and policy makers frame appropriate responses to health-related events. Nurses have begun to use social media as a voice for health care workers on the front lines. Online videos have surfaced, showing the chaos of hospital wings, and firsthand accounts of the traumas and struggles nurses have faced have appeared on sites such as Facebook [10,13].

Nurses are using social media in order to communicate with the public and advocate for more supplies and support [13]. For example, the “#GetMePPE” hashtag on Twitter was generated in order to spread awareness of the PPE shortages; this led to creation of a petition with over 62,000 signatories, which combined with a website, GetUsPPE.org, to allow health care workers to obtain hundreds of thousands of articles of PPE [14]. The COVID-19 outbreak has resulted in a set of studies that have examined public perceptions, thoughts, and concerns about this pandemic through the use of social media data. All of these studies relied on data from public digital media, such as Twitter or Weibo platforms; these studies analyzed data from early periods of the pandemic, using different sentiment analysis techniques on the general population, irrespective of users’ professions, or evolution of sentiments over time on temporal events. In this study, we specifically analyzed social media discussions by nursing professionals participating in publicly formed Facebook groups to develop longitudinal insights related to the pandemic’s impacts in terms of what health care providers experienced over time.

### Study Aims

The primary aim of this study was to explore nurses’ work experiences in dealing with the coronavirus pandemic and how it affected their emotional state. To achieve this, we specifically employed sentiment analysis, topic modeling, and information retrieval techniques to estimate the influence of physical, mental, and psychosocial factors of nurses related to the COVID-19 pandemic. The analysis captured major themes presented by the nurses who participated in publicly available social media groups from March to the end of November 2020. The analysis examined comments made to the posts as well. Specifically, we analyzed the major topics of concern that were posted by nurses (eg, lack of masks, PPE, and ventilators; fear of being infected; family difficulty; and worrying about employment). The major topics were identified, guided by findings presented in recent publications. The analysis also focused on how these topics changed over time (eg, from medical equipment shortages at the beginning of the pandemic to treatment in later stages). In addition, using a sentiment analysis technique, we analyzed the feelings and emotions, both positive and negative, expressed in the posts and comments.

This study was reviewed by the University of Massachusetts Lowell Institutional Review Board (IRB) and was determined to be exempt from review.

### Social Media Analytics State of the Art

Various approaches were used for text sentiment extraction by researchers, which can be divided into four categories: keyword, lexicon, machine learning, and hybrid. Some researchers also used linguistic rule-based methods [15], keyword-based methods [16], emotion-based models [17,18], natural language processing (NLP) [19], and case-based reasoning [20]. Keyword-based methods detect sentiment by looking for a match between words in a piece of text and emotion keywords, providing a matching index, which is also called information retrieval [16]. Lexicon-based methods use a sentiment lexicon or dictionary to detect the correct emotion from a piece of text [21]. Machine learning methods use both supervised [22,23] and unsupervised [24,25] learning for emotion detection, using various existing classification and clustering methods. Hybrid methods merge more than one of the above techniques and apply the results to recognize text emotion [16,26-28]. Emotion is generally defined and described by various emotion models. All existing emotion models can be divided into categorical and dimensional models [29]. Categorical emotion models, such as those by Ekman [30], Shaver [31], and Oatley [32], categorize all human emotions into a few major classes (eg, anger, disgust, fear, joy, love, positive, and negative). In contrast, dimensional sentiment models, such as Plutchik’s model [33], the circumplex model [34], the cognitive structure of emotions model [35], and Loveim’s model [34], classify sentiment in detail, using multiple dimensions (ie, valence, arousal, and dominance) and intensities (ie, basic, mild, and intense) in a question-and-answer form. We used the most popular methods from the existing literature—the information retrieval technique (keyword), predefined dictionary-based Linguistic Inquiry and Word Count (lexicon), and pretrained Bidirectional Encoder Representations from Transformers (BERT; machine learning) [36]—to identify sentiments in various use cases.

## Methods

### Overview

**Figure 1:**
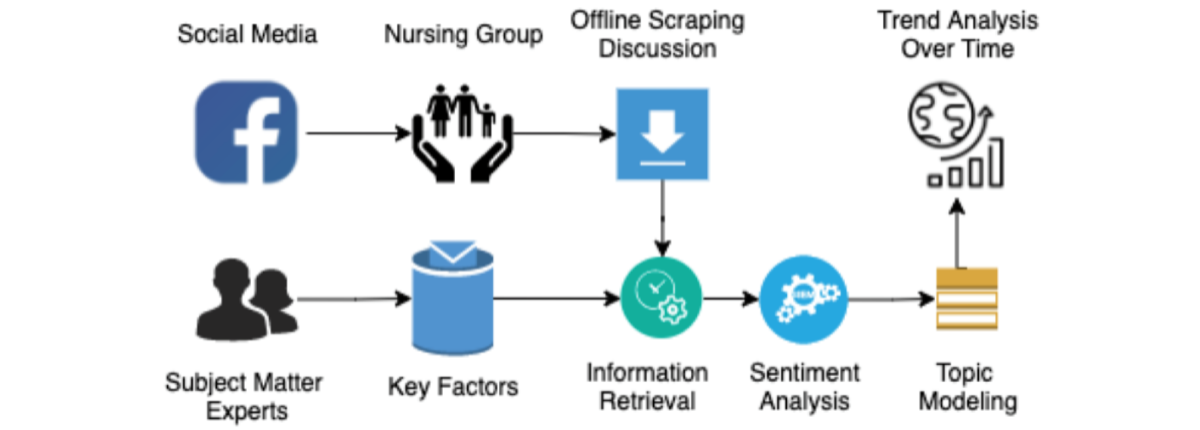
Methodology flowchart.

Social media refers to digital platforms where people can express their ideas, providing easy access to a diverse population all over the world. In particular, as of November 2020, Facebook, with 2.7 billion monthly active users, is the largest platform and plays a dominant role in social networks. In this study, we applied data mining techniques with added quotes to understand nursing professionals’ perspectives regarding the COVID-19 pandemic as discussed in trusted open Facebook groups of nurses. Figure 1 illustrates the flowchart of our methodology.

### Data Preparation

Our data preparation phase consisted of group selection, data collection, and preprocessing. First, we selected a public nursing professionals’ group, which consisted of 108,354 members, formed by nursing professionals with the sole purpose of COVID-19–related discussion, and we collected the nurses’ posts. Data collection from Facebook group posts is more challenging than the use of any other similar social media platforms, such as Twitter. Since Facebook’s application programming interface (API) lacks the capability to extract comments and other necessary information (eg, reactions and photos), we used the Facebook HTML page offline downloader and parsed the HTML tags using the Beautiful Soup library from Python (Python Software Foundation) to extract the following information: various post IDs, hash value of usernames (deidentified), post text, number of likes, date, and the comments for each post. To represent the emotion pattern during the pandemic situation, we collected posts from the beginning of the pandemic, on March 1, 2020, until November 30, 2020, and saved them in a relational database of two tables—main posts and related comments—with appropriate private-public keys definitions. The collected raw data contained background noise, such as URLs, hashtags, emoji, stop words, and empty posts, which was removed from the data using Python-based data cleaning tools in order to provide increased precision scores.

### Data Analysis Tools

We used two different analytical tools to analyze collected data: the sentiment analysis tool and the information retrieval tool.

#### Sentiment Analysis Tool

Sentiment analysis is used as a process to determine the character of a text (ie, positive, negative, or neutral), assisting one to understand overall perceptions regarding a topic of conversation. BERT is a transformer-based machine learning technique for NLP pretraining developed by Google to extract sentiments. We initially trained and validated our BERT-based supervised model on an existing Twitter data set of 1.6 million items [37]. The Twitter data set has four labels: joy, sadness, anger, and fear. For this research study, we used the BERT [36] framework to extract sentiments from the selected data texts.

#### Information Retrieval Tool

Information retrieval is a process of getting information or phrases out of the document repository. More specifically, the information retrieval tool returns texts from the database that consist of the information queried by users in the form of texts, sentences, or phrases, in order to represent top ranking or similarity scores. For this research study, we used the Python-based information retrieval tool Whoosh (Anserini), which can take either phrases, words, or documents of text or a set of conditional phrases, connected with the “and/or” relation, and return related posts of existence of queried phrases with confidence (Multimedia Appendix 1) [38].

### Validity of Choosing Analytic Tools

#### Overview

To analyze the nursing professionals’ perspectives of the COVID-19 outbreak (ie, nurses’ psychology [39,40], decision making [41,42], emotions [43], and concerns [44]), we applied current social media text analytic techniques [44,45]. In this section, we explain the validity of selecting BERT for sentiment analysis and Whoosh for information retrieval tools.

#### Bidirectional Encoder Representations From Transformers

NLP is one of the most cumbersome types of machine learning methods in the area of data preprocessing. Apart from the preprocessing and tokenizing of text data sets, it takes a great deal of time to train successful NLP models. In 2018, a team of Google scientists proposed and open-sourced BERT, a major breakthrough that took the deep learning community by storm because of its incredible performance. BERT is a transformer-based machine learning technique for NLP pretraining methods [36,46]. As per a Google scholarly citation, which has been cited over 21,000 times, BERT has been considered the most popular sentiment analysis tool for use with social media posts. There are two pretrained general BERT variations: (1) BERT-Base—a 12-layer, 768-hidden, 12-head, 110-million–parameter neural network architecture, and (2) BERT-Large—a 24-layer, 1024-hidden, 16-head, 340-million–parameter neural network architecture. Both of the BERT models have been trained on English Wikipedia (2500 million words) and BookCorpus (800 million words) and achieved the best accuracies for some of the NLP tasks, such as sentiment analysis [47,48]. In this paper, we used a pretrained BERT model proposed by Dai et al for extracting sentiments from social media posts [49]. This particular model used the original vocabulary of BERT-Base as its underlying word piece vocabulary and used the pretrained weights from the original BERT-Base as the initialization weights. Then, the model used English tweets from September 1 to October 30, 2018, to pretrain the BERT-Base model on a total of 60 million English tweets, consisting of 0.9 billion tokens. This particular BERT model achieved remarkable accuracies on sentiment analysis (>91% accuracy on Twitter posts) and fake news detection (>98% accuracy on Twitter posts), which inspired us to choose this pretrained model for our study [49] (Table 1).

**Table 1:** Performance of our pretrained BERT model compared with another model.

Target text type		BERT^a^-Base model, %	Pretrained BERT model on target, %
**Tweets**			
	Precision	89.9	91.7
	Recall	89.4	91.1
	F1 score	88.0	89.5
**Forum posts**			
	Precision	92.6	93.8
	Recall	92.4	93.4
	F1 score	92.2	93.0

^a^BERT: Bidirectional Encoder Representations from Transformers.

#### Anserini Tool

Anserini is an open-source software toolkit for the Lucene-based search engine via information retrieval toward building real-world search applications [38]. The Lucene-based search engine (Apache Lucene), first proposed in a Lucene4IR paper [50] and later improved by Grand et al [51] and Kamphuis et al [52], is widely used and is a standard foundation for search applications. The central purpose of the Anserini engine is to provide ranking (ie, indexes) of documents and sentences based on searched expression. The core components of the Anserini architecture are a multi-threaded indexing engine or wrapper, a streamlined information retrieval evaluator, and a relevance feedback engine. The wrapper provides abstractions for document collections as well as implementing an efficient, high-throughput, and multi-threaded indexer that takes advantage of these abstractions. The evaluator develops a multistage ranking architecture by extracting document features from the abstraction. The feedback component develops a relevance feedback index based on a vocabulary mismatch method between searched expressions and document collections. The final output index represents the ranking of similarity index values, where a higher value means greater similarity. We used Anserini for identifying the existence of COVID-19–related key information from the social media posts [38].

### Data Analysis

We used state-of-the-art NLP for cleaning, topic modeling, sentiment analysis, and information retrieval. During the data cleaning step, we removed background noise, such as URLs, hashtags, emoji, stop words, and empty posts, from the entire data set to increase the precision score. Then, we used BERT for detecting sentiments. In this process, we used Hugging Face’s transformer library written in TensorFlow to label our collected data with sentiments, along with the frequency [53]. Hugging Face is a Python-based transformer library that can support our pretrained BERT model and can be used to label any collected data with sentiments. This library specifically shows the potential sentiments from a text, and one needs to confirm the sentiments from an interface. One graduate student was engaged to confirm the sentiments from the Hugging Face interface. It should be mentioned that one or more sentiments can be associated with a single post; thus, a single post can be associated with multiple sentiments. In that case, a single post can be considered multiple times for multiple sentiments. These detected sentiments were used and subdivided into subtopics later. After getting the emotions measure, we explored additional topics (eg, lack of masks, PPE, and ventilators; fear of being infected; family difficulty; and worrying about employment), which are specific and cannot be detected or retrieved by use of sentiment analysis or topic modeling methods. Therefore, we used an information retrieval technique (Anserini) to further label posts. Anserini is a Python-based search engine, similar to Lucene search indexing. This will result in the posts on these topics along with the score, which is the term frequency–inverse document frequency for the topic [38]. Based on the emotional themes identified, specific anonymized posts and comments were included in this paper to highlight qualitative examples of nurses’ own words.

## Results

### Overview

The following results illuminate the negative and positive emotions expressed by nurses over time. The emotions are related to a variety of identified nurses’ experiences during a 9-month period (ie, March 1 to November 30, 2020) of the COVID-19 pandemic. Sample data (ie, comments and posts) are displayed in Table 2.

**Table 2:** Distribution of posts and comments over 9 months in 2020.

Month	Posts^a^ (n=1548), n (%)	Comments^a^ (n=109,445), n (%)
March	8 (0.5)	1739 (1.6)
April	7 (0.5)	1939 (1.8)
May	64 (4.1)	11,432 (10.4)
June	111 (7.2)	11,777 (10.8)
July	144 (9.1)	16,627 (15.2)
August	457 (29.5)	16,553 (15.1)
September	218 (14.1)	24,274 (22.2)
October	178 (11.5)	8313 (7.6)
November	361 (23.3)	16,791 (15.3)

^a^There were a total of 110,993 posts and comments combined.

### Detection of Negative Emotions Expressed by Nurses Over Time: Anger, Anxiety, and Sadness

**Figure 2:**
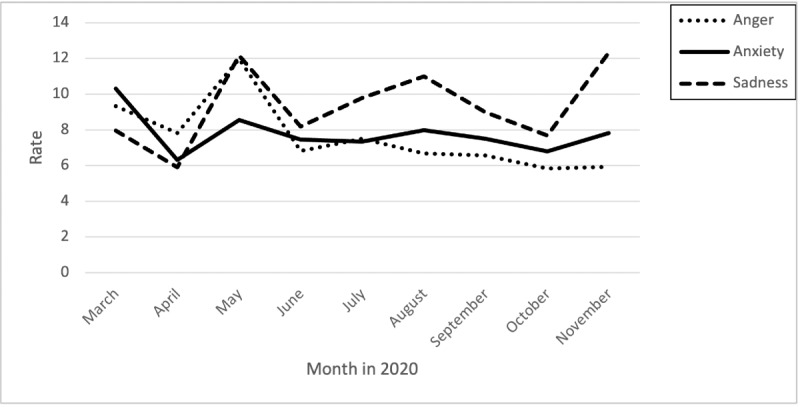
Rates of posts and comments related to the emotions of anger, anxiety, and sadness over time. The rate was calculated by dividing the number of specific posts and comments of the expressed emotion by the overall emotional posts and comments for the month.

Figure 2 shows the rate variation among the emotions of sadness, anger, and anxiety. The rate was calculated by dividing the number of specific posts and comments of the expressed emotion by the overall emotional posts and comments for the month. The displayed trend demonstrates that rates of all emotions (ie, posts and comments) peaked in May, July, and August. Sadness and anxiety rates showed an additional peak in November, while the rate of anger was trending down (Figure 2).

Sample posts and comments that exemplify these emotions are shared in this section. One nurse posted the following comment that displays anxiety with her role during the pandemic:

I am terrified we will end up hospitalized or dead. My chest feels tight, but I think it’s anxiety and not a COVID symptom.

One nurse posted a comment that demonstrates anger related to undefined policies on returning to work after exposure to COVID-19:

I am so mad they let a nurse who is COVID positive back to work with no rescreening done. He gets two hours into shift and has to go home sick. Thanks for the exposure people 

One nurse shared her feelings about depression and unhappiness, which led to reconsidering her profession as a result:

...This pandemic is absolutely draining and has even made me reconsider nursing. I am currently making a slight change and jumping into resource nursing. I’ve worked the COVID ICU [intensive care unit] now for months and have noticed myself progressively becoming more depressed and unhappy. I’m making this change for mine and my family’s sanity...

### Frustration Due to Mask Side Effects, Shortage of PPE, Media Misinformation, and Lack of Compliance With Masks

**Figure 3:**
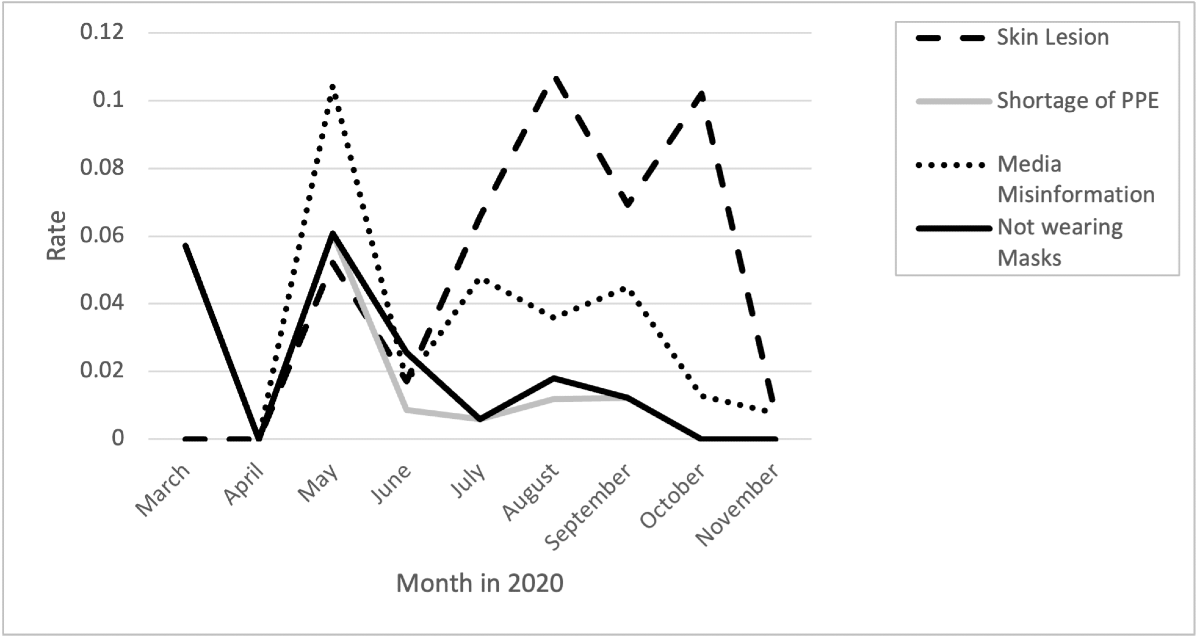
Rates of posts and comments related to personal protective equipment (PPE) and misinformation from media over time.

Frustration with misinformation from the media was ongoing, with the highest peaks from April through June. Frustration caused by shortage of PPE peaked from April through June. Frustration from skin lesions was ongoing, with the highest peaks in August and October. Frustration due to people not complying with mask recommendations peaked from April through July and again from July through September (Figure 3).

The following posts and comments illuminated the emotions expressed as they relate to frustration related to skin lesions, shortage of PPE, misinformation from media, and people not complying with mask recommendations.

Nurses posted comments about their struggle with mask rashes and lesions caused by wearing masks all day:

I’m starting to get pressure ulcer on the tip of my nose from 12 hr shifts w surgical masks on...

Nurses also reported receiving improper PPE:

They gave us surgical masks and then when COVID probable, they didn’t give us N95s until cases were exponentially increasing. FYI (I had my own N95 and brought it). Then they gave us these N95s that were not fitted - that broke when I was using it (I had to staple the straps)

Another nurse shared that her facility controlled their access to PPE:

Heck, our acute care hospital locks up the PPE. We have to sign it out and are only allowed 1 mask a shift

Some nurses posted comments about frustration with those who refuse to wear masks at work:

Anyone else returning from work after being sick with COVID annoyed/anxious over staff removing their mask at the nurses’ station all day long? Worried for my staff getting sick and tired of people just not caring. I get it we are all sooo tired of this but COVID is still here.

A few nurses expressed their concerns about the spread of misinformation about the virus:

...I’m so sick and tired of people with ZERO credentials and experience in the medical field telling others the virus is a hoax and wearing a mask is pointless and literally trying to convince others this virus isn’t a problem. People are so shortsighted on their little soapbox that they don’t realize PEOPLE are DYING and their constant ramming of conspiracy theories down people’s throats could be enough to convince someone this virus isn’t deadly and can get someone killed. I’m so irritated right now.

### Isolation as it Relates to Social Life, Family, and Friends

**Figure 4:**
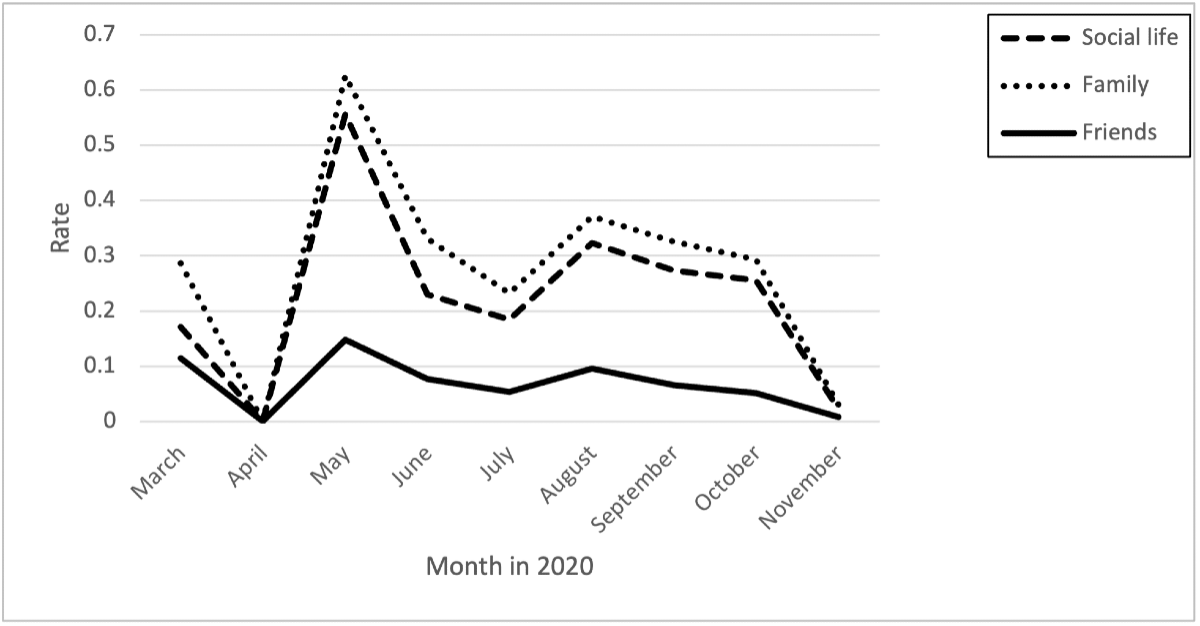
Rates of posts and comments related to isolation over time.

The rate of isolation-related posts and comments across all categories peaked across all months, with the highest rates from April through June, followed by another rate increase in July to October (Figure 4). Nurses expressed concerns about isolation from family due to fear of infecting them:

I don’t personally care about the risk to myself, it’s more the fact that I’d like to be able to see my parents again and possibly hug my mom at least once this year. She has 3 autoimmune diseases. Me being in the same room as her is a major risk.

I miss my kids. I won’t go near them. haven’t in 3 weeks. I talk to them from 10-12 feet away with a mask. It sucks.

### Exhaustion and Loneliness

**Figure 5:**
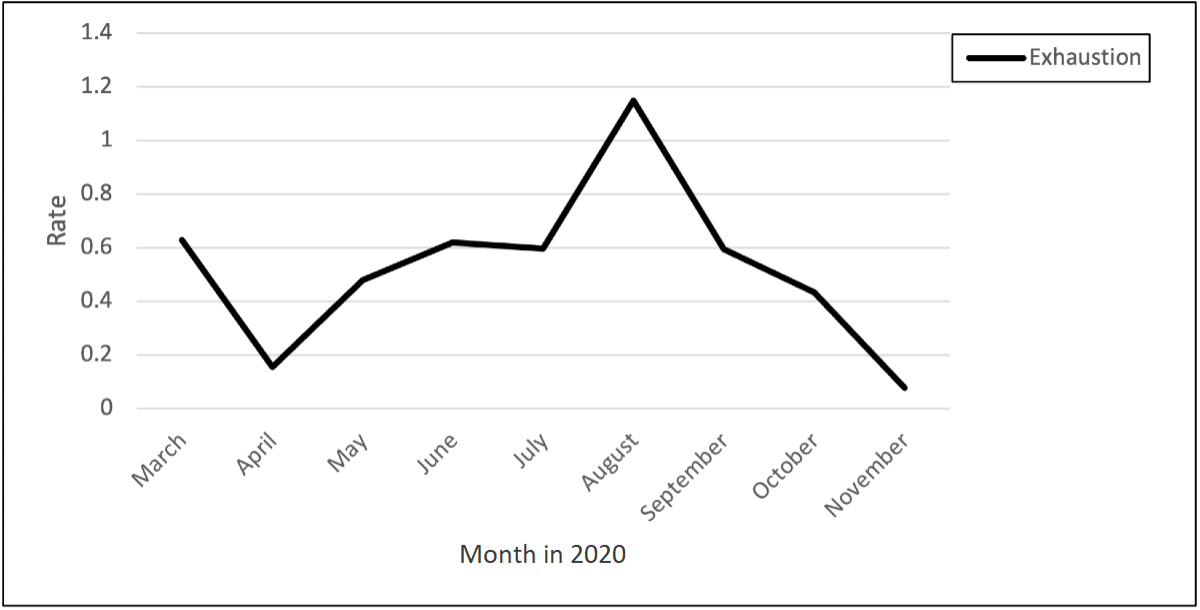
Rates of posts and comments related to exhaustion over time.

**Figure 6:**
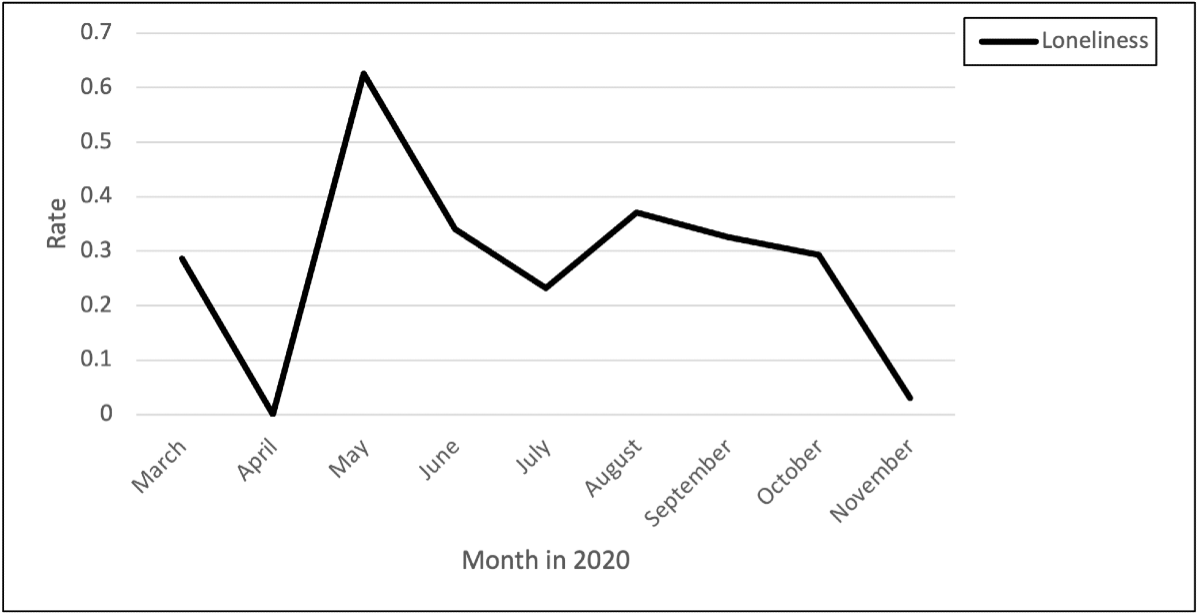
Rates of posts and comments related to loneliness over time.

The displayed trend demonstrates that exhaustion peaked over time, with a significant peak from July through September (Figures 5 and 6). Nurses described the mental and emotional exhaustion of watching patients decline from COVID-19:

Seeing these people suddenly tank and say goodbye before they get put on the vent. Face timing their loved ones, one last time, it don’t get any easier. How do you fit a lifetime of love and relationships into a 2-minute phone call? I am struggling with asking to take a break from my unit. I am exhausted mentally and emotionally.

The displayed trend demonstrates that loneliness peaked from April to the end of May, with another peak from July through October. One nurse commented on how the isolation and longing to hug their family has led them to question their career choice:

I love being a nurse and I love taking care of people; however, this pandemic has made me question my career. I’ve had a lot of time in isolation to think about it. As I long to hug my family but will only FaceTime them to keep them safe. I even had to watch my daughter’s graduation online. Is it worth it to risk my life and my families for this career?

Another nurse commented on feeling alone because of family not understanding what they are going through:

For those working third shift (like myself) how are you handling all this with your families??? Mine doesn’t understand at all.. and I feel so utterly alone right now. They tell me that I “signed up for this job” so I’m not allowed to be saddened by it. I just don’t know what to do, but I’m extremely depressed.

### Infected at Work 

**Figure 7:**
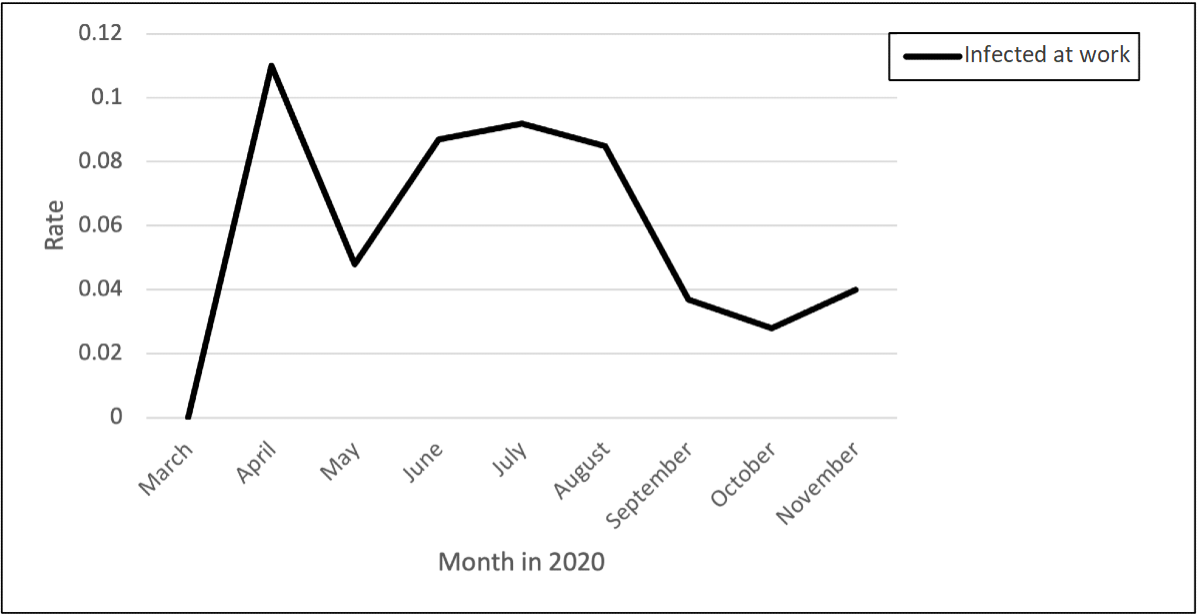
Rates of posts and comments related to fear of getting infected at work over time.

The posts and comments related to fear of getting infected peaked in April, followed by a decrease in the rate of posts and comments and another increase during June and July, after which they gradually decreased across the remaining months (Figure 7). An example post related to becoming infected at work was as follows:

We have had nearly 50 positive cases between staff and residents in our facility. No one is intentionally spreading it and we are all doing the best we can...Today was my absolute worst day ever in healthcare. I know everyone is under a lot of stress right now, but we all need to be a team. And when a teammate returns after an illness that landed them in the ER [emergency room] to go back at it and put themselves right back in the line of fire, have some compassion and show some respect!

A nurse explained how she believes she got infected from the improper PPE they were given at work:

I believe I got infected with an ill-fitting KN95 back when we had to tape them to our faces. It makes me so damn angry that the US is the “richest” country in the world and yet PPE is still a problem nine months into this crud.

Another nurse discussed how mask wearing and social distancing are not followed in break rooms:

We have a very low number of positives. We have been doing masks and social distancing where required. At work we have to wear masks in the lab but when we hit the breakroom, masks come off and no social distancing.

### Fear of Infecting Family 

**Figure 8:**
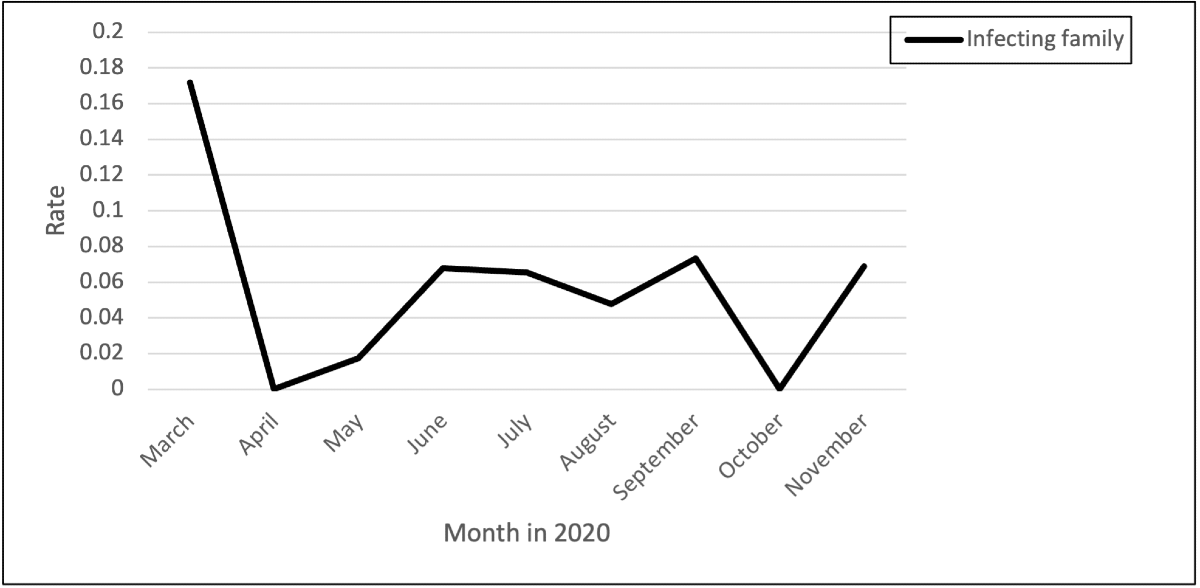
Rates of posts and comments related to fear of infecting family over time.

Nurses described the fear of infecting their family members who live in their home (Figure 8):

...I think the ultimate challenge is protecting our families. I don’t think the public totally gets the stress of how that burdens us.

Another nurse described the fear of infecting their family and others as well as having to isolate from their children:

...The increasing fear every day that I walk into the hospital that today is the day that someone who refuses to keep their mask on and coughs on me will give me the virus. I am afraid that I will be forced to self isolate and will have to explain to my small children why I can’t give them hugs and kisses, or even come upstairs. I am afraid that I will unknowingly bring it home to my family, or my next patient that I come into contact with. I am afraid that if I do just one tiny thing wrong during the donning and doffing process, that I will be the reason someone gets sick...

Another nurse explained how constantly changing protocols made her fearful of bringing COVID-19 home:

...I definitely don’t want to bring it home and infect my family. I just don’t understand why the protocols seem to differ from day to day, and even hour by hour.

### COVID-19–Positive Tests

**Figure 9:**
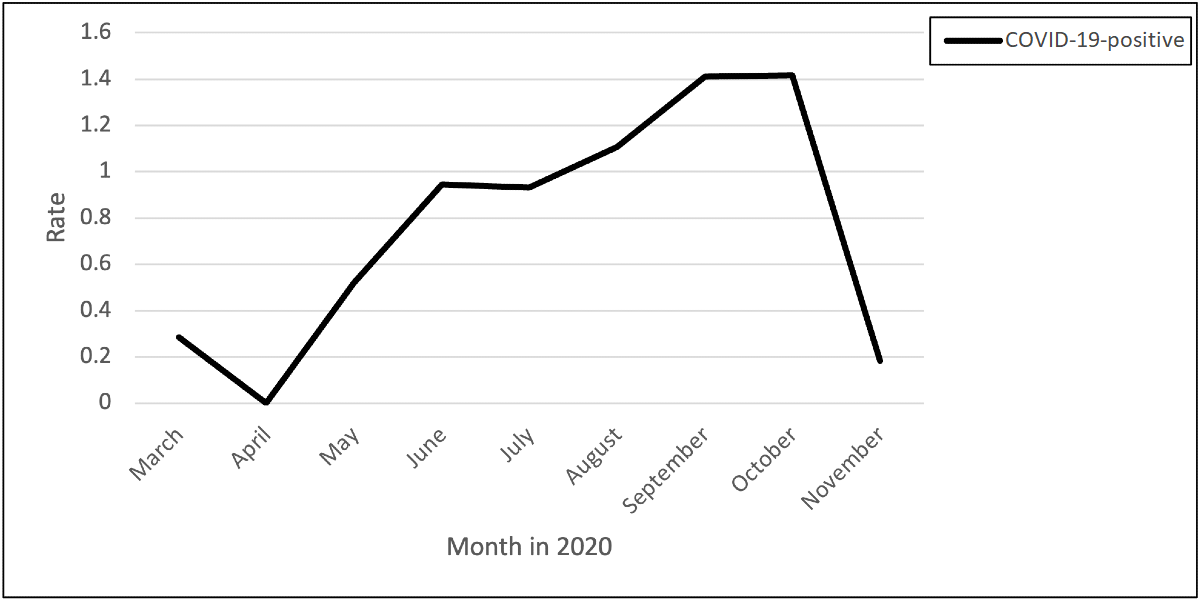
Rates of posts and comments related to testing positive for COVID-19 over time.

The rate of posts regarding testing positive for COVID-19 peaked from April through October (Figure 9). Nurses infected with COVID-19 described symptoms they experienced. One nurse posted the following:

Is there light at the end of the tunnel? On day 8 and day 6-7 I thought I was dying. Currently wondering if I should medicate for 100.5-degree temp or if it’s better to let immune system fight it. Shortness of breath is better but cough is still there along with chills, body aches, diarrhea, stress incontinence from coughing so much. I was dizzy, had numbness and pins and needles in hands and feet and did nothing but sleep for 48 hours

Symptoms from the virus lasted after recovery from the infection, compromising the ability to work:

...19 days post symptom onset, went back to work...on Sunday to work three 12s in a row after being off for almost three weeks. I am EXHAUSTED, my brain is straight fog and I move so slow. My body kills and my feet are swollen. And I’m tachy with palpitations for 90% of the night unless I’m sitting for a long period of time which does not ever happen. I don’t know how I will survive another night shift tonight. I can’t breathe in my surgical mask, let alone my n95. My chest hurts from struggling to breathe through these shifts. I know it takes time to get completely back to normal but I am so frustrated and tired ...

In addition, some of the nurses had long-lasting symptoms. One nurse posted the following in October:

I had COVID in July and my sense of smell is not anywhere back to normal. When there is an odor, I smell the most rancid smell I could ever imagine. Anyone else experiencing this?! Will I ever go back to normal?!

Exposure to the virus resulted in contracting COVID-19 and isolation from family:

...have been isolated from my family for a week. I was diagnosed last Sunday. Breaks my heart that I can’t see my children and I have to blow kisses to them from a screen. I tried my best to keep me and them safe. Praying for your health. This is no joke. I don’t wish this upon anyone.

### Paid Leave

**Figure 10:**
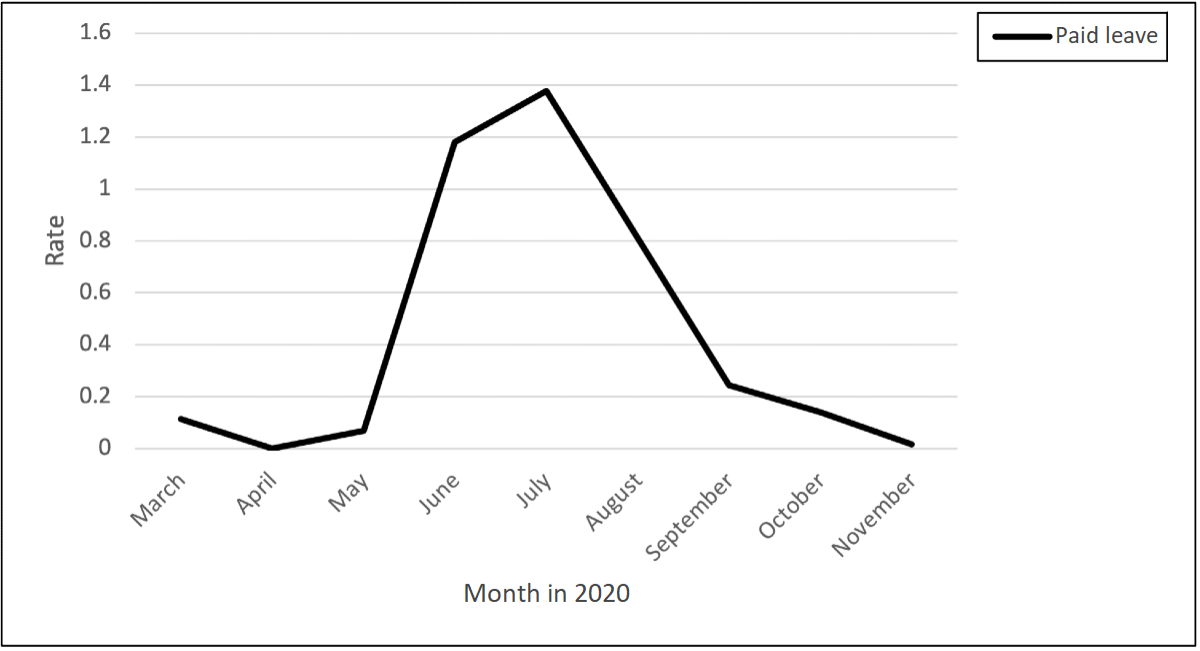
Rates of posts and comments related to paid leave due to COVID-19 over time.

The rate of posts related to paid leave peaked from the beginning of May until mid-July and then declined through November (Figure 10). Nurses posted comments about their high-risk occupation that is not reflected in hazard pay. One of the nurses posted the following:

Have been a nurse for 23 years and I agree with you. It’s a shame that we are at such high risk, and the pay truly doesn’t match the risk, not to mention the lack of pay when you finally test positive and have to stay home for 2-3 weeks (where I am now).

Many nurses were struggling with unpaid leave that added an economic burden. Variability was seen among states regarding the policy of paid or nonpaid leave for nurses who tested positive for COVID-19. One nurse posted the following:

Just tested positive. Contracted it at work...I’m now home for 2 weeks, unpaid. Can someone help me understand how this is okay...My company doesn’t have to compensate me despite contracting the virus while working. Tips? Ideas?

### Detection of Positive Emotions Expressed by Nurses Over Time

#### Patient Gratitude

**Figure 11:**
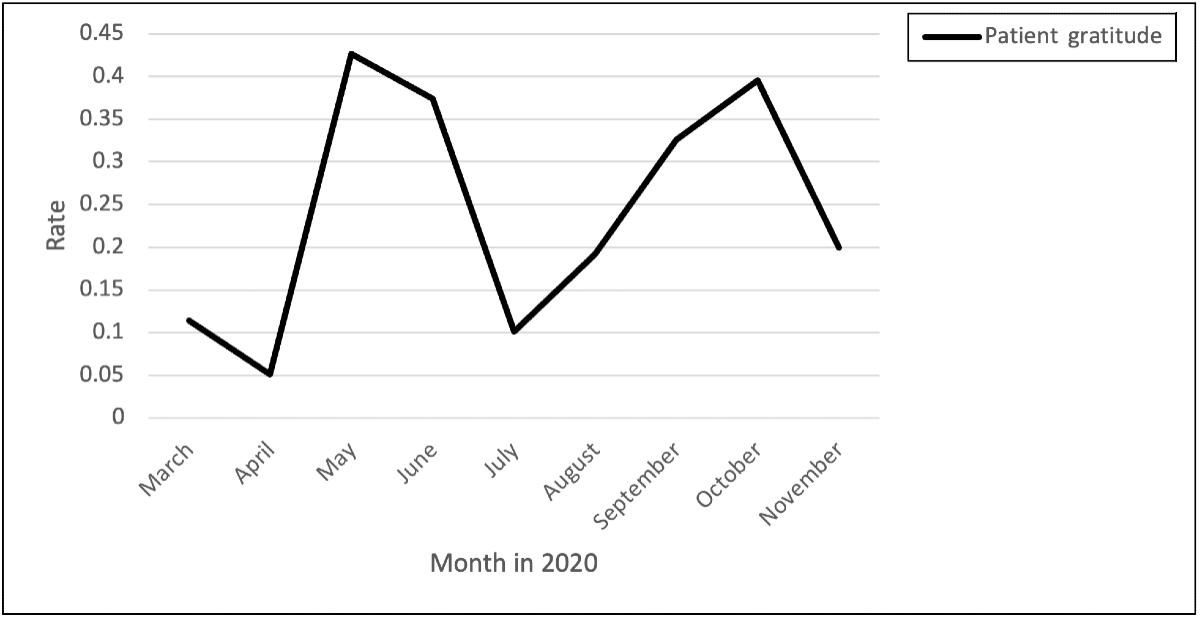
Rates of positive posts and comments related to patient gratitude over time.

Posts and comments related to patient gratitude peaked from April through June and August through October (Figure 11). One of the nurses shared an example of appreciation expressions:

We had one of our nurses have her gas paid for by another patron. I have a friend who was given a gift card to Walmart when she was shopping. And I know someone who is giving the local hospital staff certificates for a free massage as they leave work...

Another nurse commented the following:

...this makes me so happy! I’m glad some people are appreciative.

#### Hope

**Figure 12:**
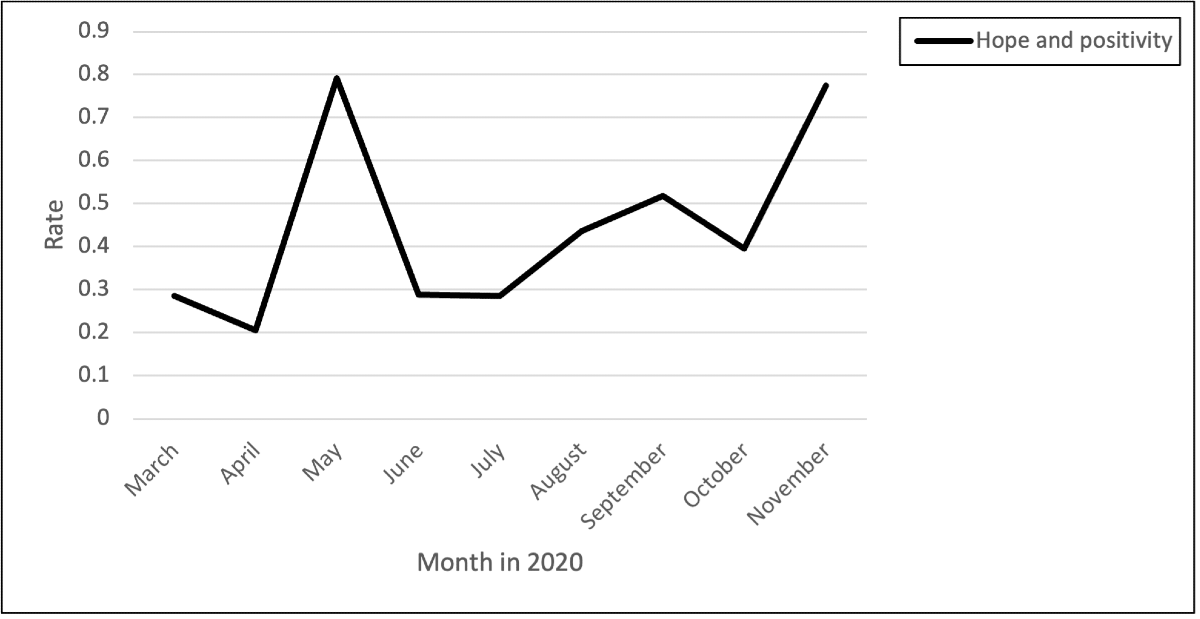
Rates of “hope” comments over time.

Expressions of hope and positivity varied across the time periods, with the highest hope and positivity in May, followed by a smaller peak in September and then a continued increase from October through November (Figure 12). One comment represents the positivity and hope expressed as it relates to the strength gained from teamwork and not going through this pandemic alone:

You are not alone. In your fear Frustration and Anger We see the tears wishing for better days You are not alone We see the strength Teamwork Desire to wrap your arms around a tired coworker Your loved ones The patient With no family near Pride in profession You are not alone We see you We are you.

Many comments reflected hope that the peak effects of the pandemic would subside and that a return to some normalcy would be on the horizon:

Hopefully someday it will be less overwhelming.

Our peak/wave is over here and hopefully never comes back.

Other comments reflected positivity as nurses encouraged each other to keep hopeful:

You will come out of it soon!...Protect yourself at all cost. Most of all keep the faith. God bless you

## Discussion

### Principal Findings

This social media study combined sentiment-detecting technology and major discussion themes to explore nurses’ emotional expressions from the beginning of the pandemic through November 2020. The analyses were supported by direct quotes illuminating the experience of being a nurse during the COVID-19 pandemic. Our data methodologies follow the standard social media text analytic literature, which has been proven effective and trustworthy and has been applied in significant social media text analytics for nursing and COVID-19 trend studies in the past [36,38-45]. Apart from that, because of the inability of Facebook’s API to extract the necessary group posts, we developed our own offline textual information extraction techniques, with appropriate deidentification to preserve users’ privacy, as per IRB exemption conditions. Combining BERT-based sentiment analytic and Anserini-based information retrieval techniques facilitated the development of a full-fledged generalized social media analytics framework that can be used in any study domain that includes, but is not limited to, perspectives of students, media personalities, social workers, and minority groups with regard to adverse social events.

Sentiments described in the posts and comments reflected a variety of negative and positive emotions toward the pandemic experience. The negative sentiments expressed by nurses were anger, specifically as it relates to undefined policies, such as returning to work after being infected; anxiety because of being a frontline worker during the pandemic; and sadness caused by witnessing patients decline and die as well as being isolated. Nurses expressed mental and emotional exhaustion. The recent literature has expressed similar sentiments. In a cross-sectional descriptive correlative study on burnout of 2014 frontline nurses in Wuhan, China, 835 nurses reported high levels of emotional exhaustion, and 556 nurses experienced high level of depersonalization [8]. Sentiments related to anger and anxiety, specifically as they relate to undefined policies during nurses’ service as frontline workers during the pandemic, were also expressed in recent studies. A commentary published by Nelson and Lee-Winn highlighted the anxiety nurses experienced as they dealt with very frequent changes in policies and protocols as the pandemic evolved [39]. Hu et al showed that 40% to 45% of frontline nurses experienced anxiety or depression, with 11% to 14% having moderate to severe anxiety or depression [8]. Results from a cross-sectional online survey of 1103 frontline emergency department nurses demonstrated that engaging in clinical services for patients with COVID-19 was significantly associated with a higher risk of depression (43.6%) [7].

Nurses shared factors that contributed to increased stress and anxiety. One example from this analysis was related to conspiracy theories and “fake news.” Recent research has supported these findings [10]. Other contributing factors to the nurses’ stress and anxiety were the shortage of PPE and noncompliance with rules on mask wearing. Nurses also reported development of skin lesions as a consequence of wearing PPE daily for long shifts. Similarly, Hu et al [8] and Shaukat et al [9] found that 1910 out of 2014 nurses had one or more skin lesions caused by PPE. Because of the shortage of PPE, the fear of becoming infected with COVID-19 as well as infecting their family members presented another factor in elevated anxiety and depression among the nurses. In addition, nurses expressed feelings of loneliness caused by isolation from their social life, family, and friends. Nelson and Lee-Win reported similar concerns. Similarly, a 2020 survey by the American Nurses Association of 10,997 nurses found that 28% felt depressed and 29% felt isolated and lonely [54].

Positive sentiments expressed by nurses were related to patient gratitude and hope as it related to teamwork and support of one another throughout the pandemic as well as hope for better days. Recent studies reported that nurses experienced positive emotions simultaneously with reporting negative emotions. There was a sense of responsibility and professional identity while they supported each other. The nurses also felt patients’ gratitude [36]. These results agreed with our findings.

The emotions described also changed over time from the beginning of the pandemic until late November 2020. The findings resemble the psychosocial and emotional responses associated with the phases of disaster as described by DeWolfe [5]. Nurses spoke about their fears and anxiety, especially as they related to their sense of loss of ability to protect themselves and others, particularly their family members. These sentiments were noted throughout the time frame of posts and comments analyzed but were heightened in the early phase of the COVID-19 pandemic. This resembles Phase 1, or the predisaster phase, and the Phase 2 impact phase of a disaster as described by DeWolfe [5]. As the COVID-19 pandemic time frame continued (Multimedia Appendix 2), emotions experienced followed the Phase 2 impact and Phase 3 heroic phases of disaster but quickly moved to the Phase 5 disillusionment phase, as in this phase, the realization of limited assistance and noncompliance of the public led to emotions of stress and burnout, with many reactions such as exhaustion, frustration, anger, and depression being exhibited in the sentiments that were expressed. As vaccines were developed and the number of cases declined, positive emotions of gratitude and hope were displayed in the sentiments of posts and comments. This resembles the Phase 4 honeymoon phase. Specifically, optimistic comments were related to patient gratitude, teamwork, and support, as well as keeping the faith that all would return to normal.

### Limitations

Although this study was designed to be a unique representation of the perspective of nurses during the COVID-19 pandemic, there is a potential error in that some posts in this group could have been made by nonnurse individuals because of open-group posting allowance. Because of the specificity of comments and group rules that were monitored by administrators, the chances of this were low and presumably would not have affected the analysis. An additional limitation to this study is that the data originated from one specific open group on Facebook and might not represent all nurses’ perspectives; however, in this one group, the membership included 106,000 nurses. In addition, findings from this study were in agreement with findings in the current published literature about this topic.

Although the authors based the analysis on the definitions described in Multimedia Appendix 3, they might not have captured all the emotions experienced by the nurses. We considered the pretrained BERT model, with four sentiment labels—joy, sadness, anger, and fear—which may slightly limit our analytic results. On the other hand, a single post that could be counted multiple times with regard to different sentiments carried the risk of introducing error into our analytic results. However, recent studies have found that considering only the above four sentiment labels and making multiple counts of the same posts for the different sentiments sustained the analytic results for COVID-19–related posts as per different machine learning techniques, which affirm the consistency of our results [55].

### Conclusions

The significance of this study is that it adds to the importance of documentation about a historical pandemic from the nurses’ experience. The COVID-19 pandemic is a unique experience that the world was not prepared for and for which we were not preparing student nurses in the nursing curriculum. Themes and information gathered from this analysis will constitute evidence of what transpired in the United States in the time of the pandemic outbreak. It will provide a voice for the nurses who served on the front line. It will also serve as a basis for articulating lessons learned and a basis for ethical discussions of other topics in health care. In addition, it will be particularly useful to various government agencies, hospitals, organizations, and communities that wish to better understand the major concerns related to crises of public health and make policies to address them.

## Appendix

Multimedia Appendix 1 Topics and their associated phrases and methods used in this analysis.

Multimedia Appendix 2 COVID-19 timeline.

Multimedia Appendix 3 Number of posts by sentiments and themes over time.

## Abbreviations

API: application programming interface
BERT: Bidirectional Encoder Representations from Transformers
ER: emergency room
ICU: intensive care unit
IRB: Institutional Review Board
NLP: natural language processing
PPE: personal protective equipment
