# Supplementary material for: Nursing Perspectives on the Impacts of COVID-19: Social Media Content Analysis
Source: JMIR Form Res. 2021 Dec 10;5(12):e31358. doi: 10.2196/31358 (PMC8668023; doi:10.2196/31358)
Supplement: Multimedia Appendix 1 [file formative_v5i12e31358_app1.docx]

Appendix 1

Table S1: Topics and the Associated Phrases /Methods Used in this Analysis

| **Theme/ Sentiment** | **Definition** | **Phrases** | **Method** |
| --- | --- | --- | --- |
| Anger | a strong feeling of displeasure and usually of antagonism [1] | Used BERT for sentiment analysis | BERT |
| Anxiety | apprehensive uneasiness or nervousness usually over an impending or anticipated ill : a state of being anxious [1] | Used BERT for sentiment analysis | BERT |
| Sadness | affected with or expressive of grief or unhappiness [1] | Used BERT for sentiment analysis | BERT |
| Mask Side Effects | The unfavorable response and adverse effect to wearing a mask | skin problem from wearing masks, Maskacne,  Acne from wearing masks, Face breakouts, cyst,  pimples, pimples around my chin | Whoosh |
| Shortage of PPE | Shortage - Lack, deficit [1]  PPE- personal protective equipment | Ranout off PPE, Limited quantity of PPE, PPE shortage, lack of affordable healthcare, without proper availability of PPE | Whoosh |
| Media Misinformation | misinformation - incorrect or misleading information[1] related to COVID -19 originated from media | Media's half-truth, Wrong about knowledge on pandemic, false reported, misinformation by all sides of media, inaccurate news, fake news,  fact check should be done by journalist | Whoosh |
| Lack of Compliance with masks | the act or process of complying to a desire, demand, proposal, or regimen or to coercion [1] of wearing a mask | without mask, not wear mask, workers not, wearing masks,  noses hanging out | Whoosh and BERT |
| Isolation | Isolate- to set apart from others [1] | It's hard to see family and friends, miss my work, isolation feeling, missing family, not able to go out, going in and out of isolation, crying alone,  keeping away from family and friends | Whoosh |
| Exhaustion | Exhausted- depleted of energy : extremely tired [1] | I was Exhausted, Burnout, I am tired, drained | Whoosh |
| Loneliness | Lonely-  a. being without company  b. cut off from others [1] | I am alone, in agony alone, isolation,  family abandoned, loneliness, helplessness | Whoosh |
| Fear of infection/ infecting family | Fear - an unpleasant often strong emotion caused by anticipation or awareness of danger [1]    Infection- the state produced by the establishment of one or more pathogenic agents (such as a bacteria, protozoans, or viruses) in or on the body of a suitable host [1]    Infect- : to contaminate with a disease-producing substance or agent (such as bacteria) [1] | Catching infection from patients, symptoms from patients, contacting patients | Whoosh and BERT |
| Covid positive | Testing positive to COVID-19 virus [1] | tested covid positive, positive and quarantined, being tested positive | Whoosh |
| Paid leave | Time away from work by an employee for which the employee received compensation [1] | paid leave, paid administrative leave, 2 weeks, paid leaves if tested positive, paid sick leave | Whoosh |
| Patients gratitude | Gratitude- the state of being grateful [1] | gratitude to express how much nurses care, thank you, patient express gratitude, thankful and grateful for work, you are super hero | Whoosh |
| Hope and positivity | to cherish a desire with anticipation : to want something to happen or be true [1]  the quality or state of being positive [1] | keep the faith, hang in there, hope helps, optimism, stay strong, believe in yourself, team work | Whoosh |
| Loss of Pay | Losing pay due to being out of work with COVID-19 | no sick leave, loss of pay, no pay if sick, no pay during apr/may | Whoosh |
| Family Support | Assist, help [1] from family members of nurses on the frontlines | Family or friends that help, happy because of family support, grateful, care and love from family | Whoosh |

**References**

1. Merriam-Webster. Merriam-Webster. https://www.merriam-webster.com/ [Accessed Nov. 21, 2021].
