# Supplementary material for: Nursing Perspectives on the Impacts of COVID-19: Social Media Content Analysis
Source: JMIR Form Res. 2021 Dec 10;5(12):e31358. doi: 10.2196/31358 (PMC8668023; doi:10.2196/31358)
Supplement: Multimedia Appendix 2 [file formative_v5i12e31358_app2.docx]

Appendix 2

Table S1: COVID 19 Timeline

| **Political/ Governmental Actions** | March 13 — Trump Declares COVID-19 a National Emergency  September 16 — Trump Administration Releases Vaccine Distribution Plan  November 9 — President-Elect Biden Announces COVID-19 Transition Team |
| --- | --- |
| **Care** | July 23 — Antibody Cocktail May Treat, Prevent COVID-19  August 4 — Rural Hotspots Face Lack of Intensive Care Unit Beds  August 23 — Convalescent Plasma Is Cleared for Use by FDA  September 3 — Steroids Reduce Mortality in Severe Cases  October 22 — FDA Approves Remdesivir as First COVID-19 Drug |
| **Vaccines** | May 21 — United States and AstraZeneca Form Vaccine Deal  July 14 — Early Moderna Data Point to Vaccine Candidate’s Efficacy  July 27 — Moderna Vaccine Begins Phase 3 Trial  September 16 — Trump Administration Releases Vaccine Distribution Plan  September 21 — Johnson & Johnson Begins Phase 3 Vaccine Trial  October 9 — US Signs Deal with AstraZeneca  Mid November — High efficacy results for Moderna and Pfizer are reported. |
| **COVID 19 Spread** | March 11 — WHO Declares COVID-19 a Pandemic  June 10 — US COVID-19 Cases Reach 2 Million  July 7 — US Surpasses 3 Million Infections, Begins WHO Withdrawal  July 9 — WHO Announces COVID-19 Can Be Airborne  August 17 — COVID-19 Now the Third-Leading Cause of Death in the US  August 28 — First Known Case of COVID-19 Reinfection Reported in the US  September 23 — A New, More Contagious Strain of COVID-19 Is Discovered  September 28 — Global COVID-19 Deaths Surpass 1 Million  October 15 — US Cases Spike Again |
