# Supplementary material for: Nursing Perspectives on the Impacts of COVID-19: Social Media Content Analysis
Source: JMIR Form Res. 2021 Dec 10;5(12):e31358. doi: 10.2196/31358 (PMC8668023; doi:10.2196/31358)
Supplement: Multimedia Appendix 3 [file formative_v5i12e31358_app3.docx]

Appendix 3

Table S1: Number of posts by sentiments and themes over time

| **Month** | March | April | May | June | July | August | September | October | November |
| --- | --- | --- | --- | --- | --- | --- | --- | --- | --- |
| Total number of posts | 8 | 6 | 63 | 103 | 129 | 438 | 211 | 177 | 355 |
| Total number of comments | 1739 | 1939 | 11432 | 11661 | 16627 | 16282 | 24322 | 7661 | 12691 |
| **Figure 2** |  |  |  |  |  |  |  |  |  |
| Sad | 97 | 51 | 733 | 532 | 1158 | 1478 | 113 | 366 | 1159 |
| Anger | 367 | 89 | 668 | 380 | 716 | 714 | 784 | 274 | 390 |
| Anxiety | 315 | 164 | 1077 | 1186 | 1803 | 2005 | 2338 | 690 | 1510 |
| **Figure 3** |  |  |  |  |  |  |  |  |  |
| Not wearing a mask | 1 |  | 7 | 3 | 1 | 3 | 3 |  |  |
| Frustration due to skin lesion | 6 |  |  | 2 | 11 | 18 | 17 | 8 | 1 |
| Frustration due to PPE shortage | 1 |  | 7 | 3 | 1 | 3 | 3 |  |  |
| Frustration due to misinformation | 1 |  | 12 | 2 | 8 | 6 | 11 | 1 | 1 |
| **Figure 4** |  |  |  |  |  |  |  |  |  |
| Isolation due to social life | 3 |  | 64 | 27 | 31 | 54 | 67 | 20 | 3 |
| Isolation due to Family | 5 |  | 72 | 39 | 39 | 62 | 80 | 23 | 4 |
| Isolation due to Friends | 2 |  | 17 | 9 | 9 | 16 | 16 | 4 | 1 |
| **Figure 5** |  |  |  |  |  |  |  |  |  |
| Exhaustion | 11 | 3 | 55 | 73 | 100 | 192 | 146 | 34 | 10 |
| **Figure 6** |  |  |  |  |  |  |  |  |  |
| Loneliness | 5 | 0 | 72 | 40 | 39 | 62 | 80 | 23 | 4 |
| **Figure 7** |  |  |  |  |  |  |  |  |  |
| Fear of getting infected by patients |  | 2 | 5 | 10 | 15 | 14 | 9 | 2 | 5 |
| **Figure 8** |  |  |  |  |  |  |  |  |  |
| Fear of infecting family | 3 |  | 2 | 8 | 11 | 8 | 18 |  | 9 |
| **Figure 9** |  |  |  |  |  |  |  |  |  |
| Tested positive for COVID | 5 |  | 60 | 111 | 156 | 185 | 346 | 111 | 24 |
| **Figure 10** |  |  |  |  |  |  |  |  |  |
| Paid leave |  |  | 8 | 139 | 231 | 136 | 60 | 11 | 2 |
| **Figure 11** |  |  |  |  |  |  |  |  |  |
| Patients’ gratitude | 2 | 1 | 49 | 44 | 17 | 32 | 80 | 31 | 26 |
| **Figure 12** |  |  |  |  |  |  |  |  |  |
| Hope | 5 | 4 | 91 | 34 | 48 | 73 | 127 | 31 | 101 |

## 
